# Local Erythropoietin Injection in Tibiofibular Fracture Healing

**DOI:** 10.5812/traumamon.7099

**Published:** 2013-01-15

**Authors:** Hooman Bakhshi, Gholamhossein Kazemian, Mohammad Emami, Ali Nemati, Hossein Karimi Yarandi, Farshad Safdari

**Affiliations:** 1 Department of Orthopedic Surgery, Imam Hossein Hospital, Shahid Beheshti University of Medical Sciences, Tehran, IR Iran; 2 Orthopedic Research Center, Akhtar Orthopedic Hospital, Shahid Beheshti University of Medical Sciences, Tehran, IR Iran

**Keywords:** Fractures, Bone, Erythropoietin, Wound Healing

## Abstract

**Background:**

Erythropoietin (EPO), in addition to its function as an erythropoiesis regulator has a regenerative activity on some nonhematopoietic tissues. Animal studies have suggested a role for erythropoietin in bone healing.

**Objectives:**

The present study aimed to evaluate the effects of local EPO injection in healing of tibiofibular fractures.

**Materials and Methods:**

In a prospective double blind study, 60 patients with tibiofibular fracture were divided to equal EPO or placebo groups, randomly. Patients received local injection of either EPO or a placebo to the site of fracture two weeks after surgical fixation. Patients were followed by clinical and radiographic examination to determine the union rate. The period of fracture union and incidence of nonunion were compared between the two groups.

**Results:**

The demographic data and types of fractures were similar in the both groups. The mean duration of the fracture union was 2.1 weeks shorter in those treated with EPO (P = 0.01). Nonunion was observed in 6 patients of the control group and 2 receiving EPO (P = 0.02). No patient experienced any adverse effect from local EPO injections.

**Conclusions:**

EPO injection into the site of tibiofibular fractures may possibly accelerate healing.

## 1. Background

Erythropoietin (EPO) is a glycoprotein produced by the kidney which promotes the formation of red blood cells in the bone marrow. Recent studies have determined additional roles of EPO in tissue survival and cellular proliferation (1-3). It seems that EPO may accelerate bone formation and contribute to the fracture healing (1). Despite this fact, the subject is still in debate (2). Tibial fractures are of the most common fractures in orthopedics and delayed union in long bones is mostly seen in the Tibia (4, 5). Most fractures heal within 6 months. Failure of fracture healing is more probable due to limited soft tissue coverage and blood supply of the Tibia (1). Prolonged rehabilitation and delay in returning to work impose a great deal of economic pressure on patients and the society (6). These facts highlight the importance of finding ways to accelerate fracture union and diminish nonunion or delayed union.

## 2. Objectives

 Favorable outcomes of preclinical trials on efficiency and positive impacts of pharmacological products and biologic agents on accelerating fracture healing (7-9) encouraged us to design the present study to evaluate the effect of EPO on treating tibiofibular bone fractures.

## 3. Materials and Methods

A randomized double blind clinical trial consisting of 60 patients with tibiofibular fractures referred to our clinic from 2007 to 2010 was designed. Patients with multiple lower extremities, metaphyseal and isolated tibial, pathologic, comminuted, osteoporotic and open fractures were excluded. Also patients under treatment with steroids, anticoagulants, NSAIDs, calcium channel blockers and nicotine were excluded from the study. All patients underwent surgical fixation by close intramedullary nailing. The participants were divided into two groups randomly. EPO (4000 IU) 3 was injected in one group and placebo in the other into the fracture site two weeks after the operation and under sterile condition and guide of C-arm. The patients were followed afterwards by physical examination and radiographic imaging every forth night until union was confirmed. Radiographic criteria of union were callus formation in 3 of 4 cortices and clinical criteria were stability and lack of pain upon pressure over the fracture site. If the fracture was not completely healed after 9 months, it was considered as nonunion. To heed the ethics, we followed Goldhahn et al. protocol of transition from preclinical to clinical trials. Informed consent was taken from all patients prior to being included in the study. The accumulated data was statistically assessed using version 18.

## 4. Results

We followed 60 patients with simultaneous fractures of the tibia and fibula divided into two groups of 30, treated either with EPO or placebo. The mean age of groups was 31.7 ± 11.6 and 31.1 ± 11 years respectively (P = 0.52). The EPO group was comprised of 26 (86%) males and 4 females and the other group 28 (93%) males and 2 females. In the EPO group, there were 17 cases with oblique fractures, 7 transverse and 6 spiral while in the other group were 12, 10 and 8 respectively. According to the radiographic studies the mean union period was 19.35 ± 2.66 weeks in the EPO group compared to 21.50 ± 3.18 in the placebo group (P = 0.01). Two patients in the EPO group and 6 in the control group showed nonunion (P = 0.02).We detected 1 case of infection in the control group. No patient experienced any adverse effect on local EPO injection afterwards.

## 5. Discussion

Finding ways to accelerate union and diminish nonunion or delayed union is among interest in orthopedic surgery. Patients who were treated with local EPO injection experienced a two week faster union and lower nonunion rate in our study. The BESTT research team designed a prospective, randomized and single blind study to evaluate safety and efficacy of 4 recombinant human bone morphogenic protein 2 (rhBP2) in open tibia shaft fractures. They demonstrated a lower need for bone grafts in patients who received standard treatment plus implant containing 1.5 mg/ml of rhBMP2 (10). Swiontkowski and colleagues studied the effects of rhBMP2 in patients with open tibia fractures in 2006. In their trial, the control group was treated by intramedullary nailing along with other traditional methods used to treat soft tissue damage, while in the case group they used rhBMP2 alongside other methods. They reported that rhBMP2 can considerably decrease the need for bone graft surgeries in type 3 tibia open fractures (11). In 2007, Holstein et al. studied the effects of EPO on closed femoral fractures in mice. They reported that callus formed resisted more strongly against forces of torsion in those that received EPO and concluded that EPO can accelerate endochondral ossification processes and facilitate transformation of soft callus to a hard one (1). Angiogenesis plays a critical role during growth, maturation and repair phases of musculoskeletal system. Consequently, bone formation is impossible without vascular interactions (12, 13). There are two important hormonal pathways to stimulate angiogenesis: vascular endothelial growth factor (VEGF) pathway and Angiopoietin; the former is more important (10). VEGF, a signal protein, produced by cells that stimulated angiogenesis, is quite similar to EPO. Recent studies have shown that EPO receptor interaction is not limited to hematopoiesis and EPO receptors were expressed in some non-hematopoietic cells such as vascular endothelial, as well (2). Accordingly, it is possible that VEGF positive effects on fracture healing, especially on osteogenesis could be initiated with EPO as well. In this trial, patients tolerated EPO injection well and showed no side effects. Another important finding was significant lower rates of nonunion in the EPO 5 group. This finding alone may point out the important and effective role of EPO in accelerating fracture healing. We should emphasize that our knowledge about these agents and their various effects on humans are very limited and we need additional studies before we can actually use them. We recommend more studies to be designed to find the effective safe dosage of the drug as well as to choose the proper injection method, systemic versus local; and to assess the EPO effect on known cases of nonunion. It seems that local injection of EPO may accelerate fracture union and diminishes the nonunion rate of tibiofibular fractures.
